# Supplementary material for: Clustering individuals using INMTD: a novel versatile multi-view embedding framework integrating omics and imaging data
Source: Bioinformatics. 2025 Mar 22;41(4):btaf122. doi: 10.1093/bioinformatics/btaf122 (PMC11978392; doi:10.1093/bioinformatics/btaf122)
Supplement: btaf122_Supplementary_Data [file btaf122_supplementary_data.docx]

# Supplementary methods

## Computational complexity of INMTD

Here we will compute the computational complexity of the whole procedure of INMTD. First of all, initializing $G_{1}$ and $G_{2}$ involves running SVD on $X_{12}$ once, $G_{3}$ involves SVD on $\mathcal{X}_{134}$ for $p_{4}$ times, and $G_{4}$ involves SVD on $\mathcal{X}_{134}$ for $p_{3}$ times, resulting in a complexity of $O(\text{min(}p_{1}p_{2}^{2}\text{,}p_{1}^{2}p_{2}\text{)}+p_{4}\text{⋅min(}p_{1}p_{3}^{2}\text{,}p_{1}^{2}p_{3}\text{)+}p_{3}\text{⋅min(}p_{1}p_{4}^{2}\text{,}p_{1}^{2}p_{4}\text{))}$. If we assume $p_{1}\geq p_{2},p_{3},p_{4}$, it can be simplified to $O\left( p_{1}p_{2}^{2}+p_{1}p_{4}p_{3}^{2}+p_{1}p_{3}p_{4}^{2} \right)$. Then, the complexity of initializing $S_{12}$ and $\mathcal{S}_{134}$ is $O(p_{1}p_{2}r_{1}+p_{1}p_{3}p_{4}r_{1})$ based on formula (12) and (13). Hence, the initialization step is linear to the number of subjects, $p_{1}$. Subsequently, the multiplicative updating of $G_{1}$, $G_{2}$, $G_{3}$, $G_{4}$, $S_{12}$ and $\mathcal{S}_{134}$ per iteration takes $O(p_{1}^{2}\left( r_{1}+p_{2}+p_{3}p_{4} \right))$, $O(p_{1}p_{2}r_{1})$, $O(p_{1}r_{1}r_{3}r_{4}+p_{1}p_{3}p_{4}r_{3})$, $O(p_{1}r_{1}r_{3}r_{4}+p_{1}p_{3}r_{3}r_{4}+p_{1}p_{3}p_{4}r_{4})$, $O(p_{1}r_{1}^{2})$, and $O(p_{1}p_{3}p_{4}r_{1}+p_{1}r_{1}^{2})$, respectively, assuming $p_{1}\geq p_{2},p_{3},p_{4}$ and $r_{1}\geq r_{2},r_{3},r_{4}$. Summing up the initialization and training steps, the total computational complexity of running INMTD is $O(t\cdot p_{1}^{2}\left( r_{1}+p_{2}+p_{3}p_{4} \right))$, where $t$ refers to the number of iterations.

## Simulation procedure

The simulation aims to generate two nonnegative datasets $X_{12}\in\mathbb{R}_{+}^{p_{1}\times p_{2}}$ and $\mathcal{X}_{134}\in\mathbb{R}_{+}^{p_{1}\times p_{3}\times p_{4}}$ that share the same $p_{1}$ samples. To mimic the co-cluster structure of real-life data, every dimension $p_{1},p_{2},p_{3},p_{4}$ has a rank (number of clusters) $r_{1},r_{2},r_{3},r_{4}$, respectively, where $p_{i}\gg r_{i}, i=1,2,3,4$. We first simulate the cluster membership for each dimension, $C_{i}\in\mathbb{R}_{+}^{p_{i}}$, via random sampling from $\left\{ 1,2,\cdots,r_{i} \right\}$, where $i=1,2,3,4$. We then simulate the embedding matrix of each dimension to be the one-hot encoded matrix of the corresponding cluster membership vector with random noise:

$$G_{i}=\text{onehot}\left( C_{i} \right)+U\left( 0,1 \right), i=1,2,3,4$$

where $G_{i}\in\mathbb{R}_{+}^{p_{i}\times r_{i}}$, and $U\left( 0,1 \right)$ is the uniform distribution from 0 to 1. To make the embedding values resemble probabilities, where values within $[0.5,1]$ indicate membership to a cluster and values within $[0,0.5]$ indicate non-membership to a cluster, we rescale values in $G_{i}$ to $[0,1]$ via a sigmoid function:

$$\hat{G}_{i}=\frac{1}{1+e^{-{(G_{i}-1)}/{0.1}}}, i=1,2,3,4$$

where all operations are element-wise.

Subsequently, we simulate the core matrix $S_{12}\in\mathbb{R}_{+}^{r_{1}\times r_{2}}$ and core tensor $\mathcal{S}_{134}\in\mathbb{R}_{+}^{r_{1}\times r_{3}\times r_{4}}$ from uniform distribution $U(0,1)$.

The generation of $X_{12}$ is based on the reconstruction formula of NMTF:

$$X_{12}=\hat{G}_{1}S_{12}\hat{G}_{2}^{T}+U(0,1)$$

The generation of $\mathcal{X}_{134}$ is based on the reconstruction formula of NTD:

$$\mathcal{X}_{134}= \mathcal{S}_{134}\times_{1}\hat{G}_{1}\times_{2}\hat{G}_{3}\times_{3}\hat{G}_{4}+U(0,1)$$

## Comparative methods

To compare the clustering performance on the synthetic data with INMTD, we implement a few well-established or state-of-the-art methods:

$\boldsymbol{k}$**-means**: It is one of the most popular clustering methods and has close relationship with NMF. We apply $k$-means in three scenarios: 1) only $X_{12}$, 2) only $\mathcal{X}_{134}$, and 3) both $X_{12}$ and $\mathcal{X}_{134}$. The first two scenarios represent single-view clustering options and the last scenario achieves simple multi-view clustering by concatenating features from $X_{12}$ and $\mathcal{X}_{134}$. Note that $\mathcal{X}_{134}$ in both scenario 2 and scenario 3 is flatten into a 2D matrix $X_{134}\in\mathbb{R}_{+}^{p_{1}\times p_{3}p_{4}}$, because the classic $k$-means algorithm cannot deal with higher-order data. In all scenarios, input data is first standardized and $k$-means is run with $r_{1}$ clusters, one random set and maximal 200 iterations.

***NMTF***: It is one of the building blocks of INMTD, and therefore very worth a comparison in performance. As NMTF works on single 2D dataset, we apply it to only $X_{12}$. We use the ‘NMTF’ function in the R package ‘nnTensor’ with default settings except that we specify the ranks to be $(r_{1},r_{2})$ and maximal iterations to be 200.

***NTD***: It is one of the building blocks of INMTD, and therefore very worth a comparison in performance. As NTD works on single 3D dataset, we apply it to only $\mathcal{X}_{134}$. We use the ‘NTD’ function in the R package ‘nnTensor’ with default settings except that we specify the ranks to be $(r_{1},r_{3},r_{4})$ and maximal iterations to be 200.

***siNMF*** (simultaneous NMF): It is a multi-view clustering method based on NMF and has been widely used in many papers for application or comparison. We apply it jointly to $X_{12}$ and $\mathcal{X}_{134}$. Note that $\mathcal{X}_{134}$ is flatten into a 2D matrix because siNMF cannot deal with higher-order data. We use the ‘sinmf’ function in the R package ‘nnTensor’ with default settings except that we specify the rank to be $r_{1}$ and maximal iterations to be 200.

***jNMF*** (joint NMF): It is a multi-view clustering method based on NMF and has been widely used in many papers for application or comparison. We apply it jointly to $X_{12}$ and $\mathcal{X}_{134}$. Note that $\mathcal{X}_{134}$ is flatten into a 2D matrix because jNMF cannot deal with higher-order data. We use the ‘jnmf’ function in the R package ‘nnTensor’ with default settings except that we specify the rank to be $r_{1}$ and maximal iterations to be 200.

***SNF*** (similarity network fusion): It is a multi-view clustering method NOT based on NMF but network analysis, and has shown superior performance in many papers. We apply it jointly to $X_{12}$ and $\mathcal{X}_{134}$. Note that $\mathcal{X}_{134}$ is flatten into a 2D matrix because SNF cannot deal with higher-order data. We use the standard procedure provided by the R package ‘SNFtool’, as follows:

| Step 1: apply standard normalization (function ‘standardNormalization’) to $X_{12}$ and $\mathcal{X}_{134}$  Step 2: compute squared Euclidean distance (function ‘dist2’) between all pairs of data points in $X_{12}$ and $\mathcal{X}_{134}$  Step 3: compute similarity graph (function ‘affinityMatrix’) from $X_{12}$ and $\mathcal{X}_{134}$ with K=200 and alpha=0.5  Step 4: fuse the graphs of $X_{12}$ and $\mathcal{X}_{134}$ (function ‘SNF’) with K=200 and T=20  Step 5: apply spectral clustering to the fused graph (function ‘spectralClustering’) with $r_{1}$ clusters |
| --- |

The hyperparameters alpha and T are chosen by default. K is set to 200 based on the recommendation of the original paper, i.e. $K=N/C$, where $N$ is the number of samples and $C$ the number of clusters.

# Supplementary figures and tables


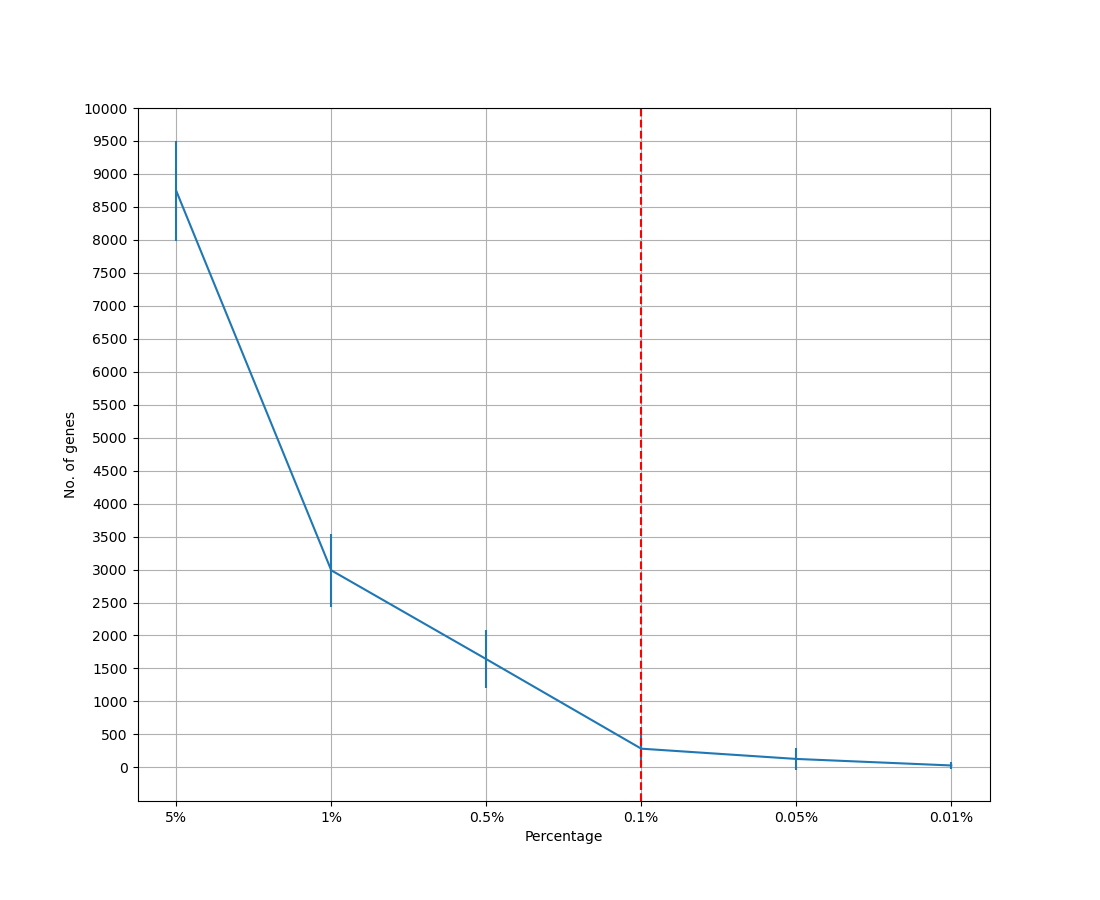


**Supp. Fig. 1: Average No. of genes (y-axis) selected for each population subgroup with different thresholds (x-axis).** The red vertical dashed line indicates the selected threshold.


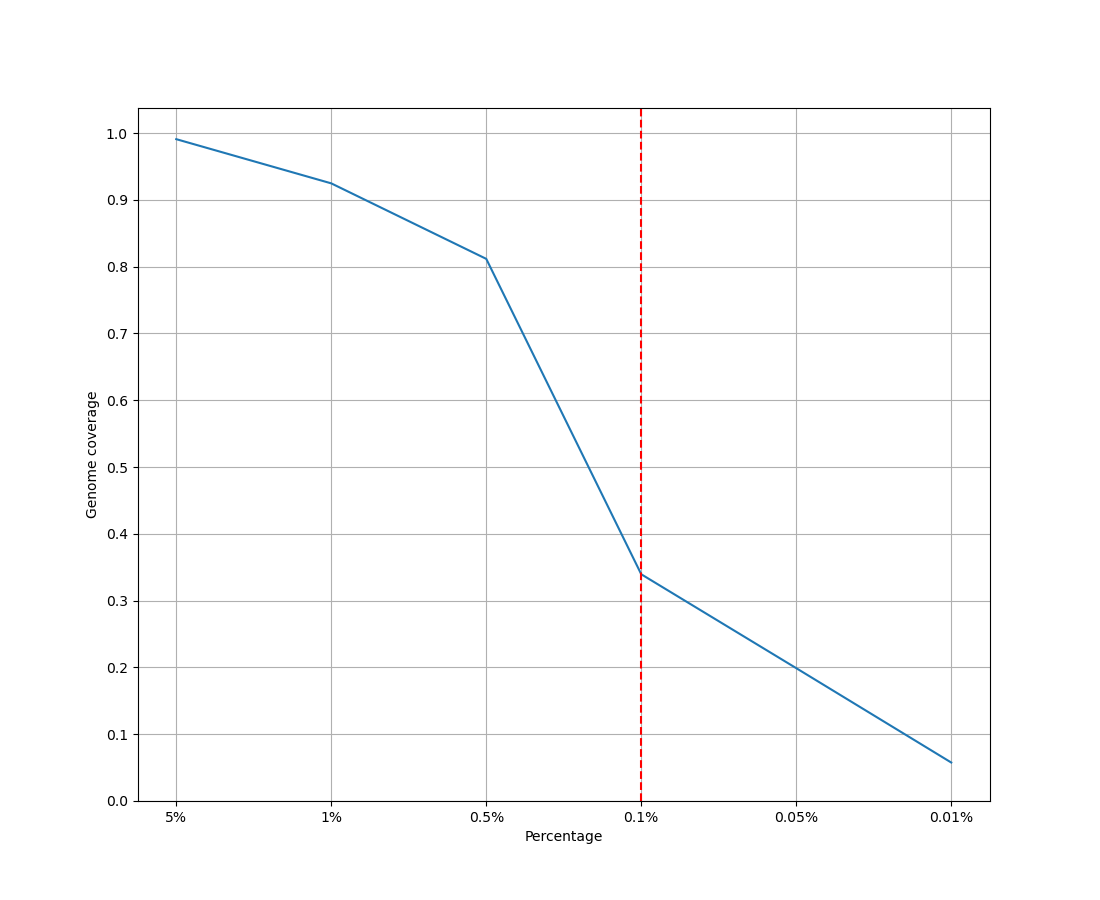


**Supp. Fig. 2: Genome coverage of genes (y-axis) selected for all population subgroups with different thresholds (x-axis).** The red vertical dashed line indicates the selected threshold.


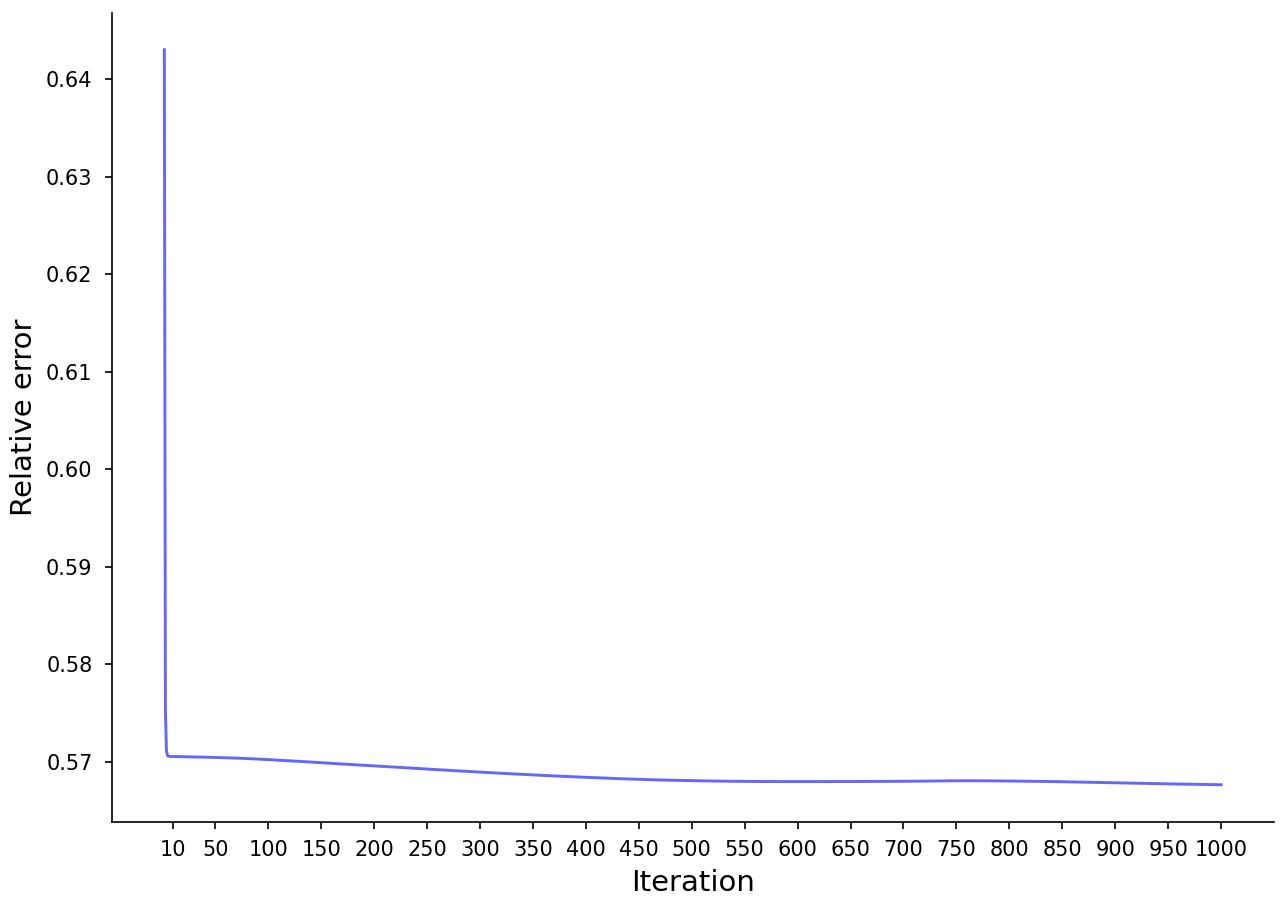


**Supp. Fig. 3. Trajectory of the total relative error.**


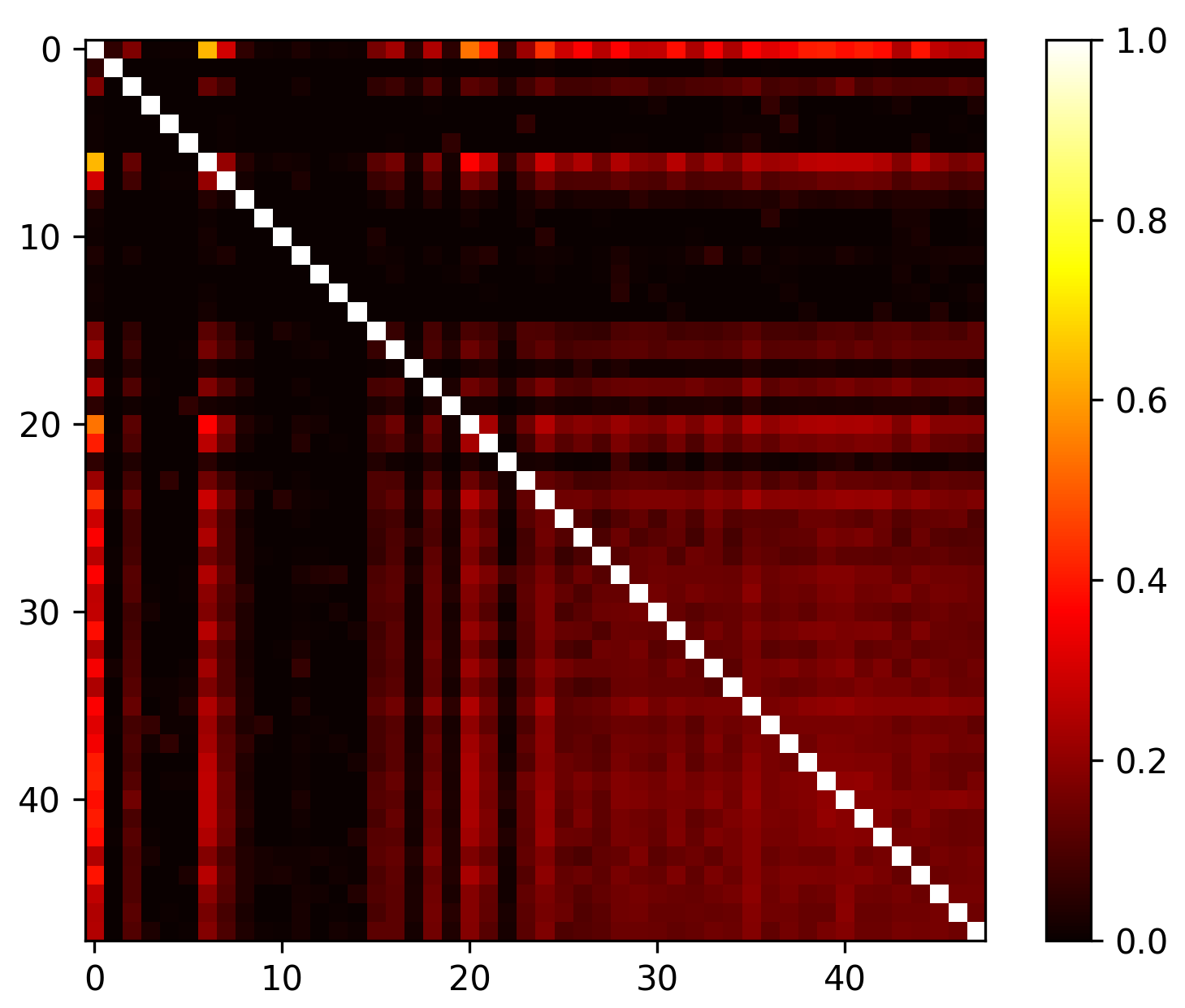


**Supp. Fig. 4: Heatmap of the covariance matrix of** $\boldsymbol{G}_{\boldsymbol{1}}$**.**


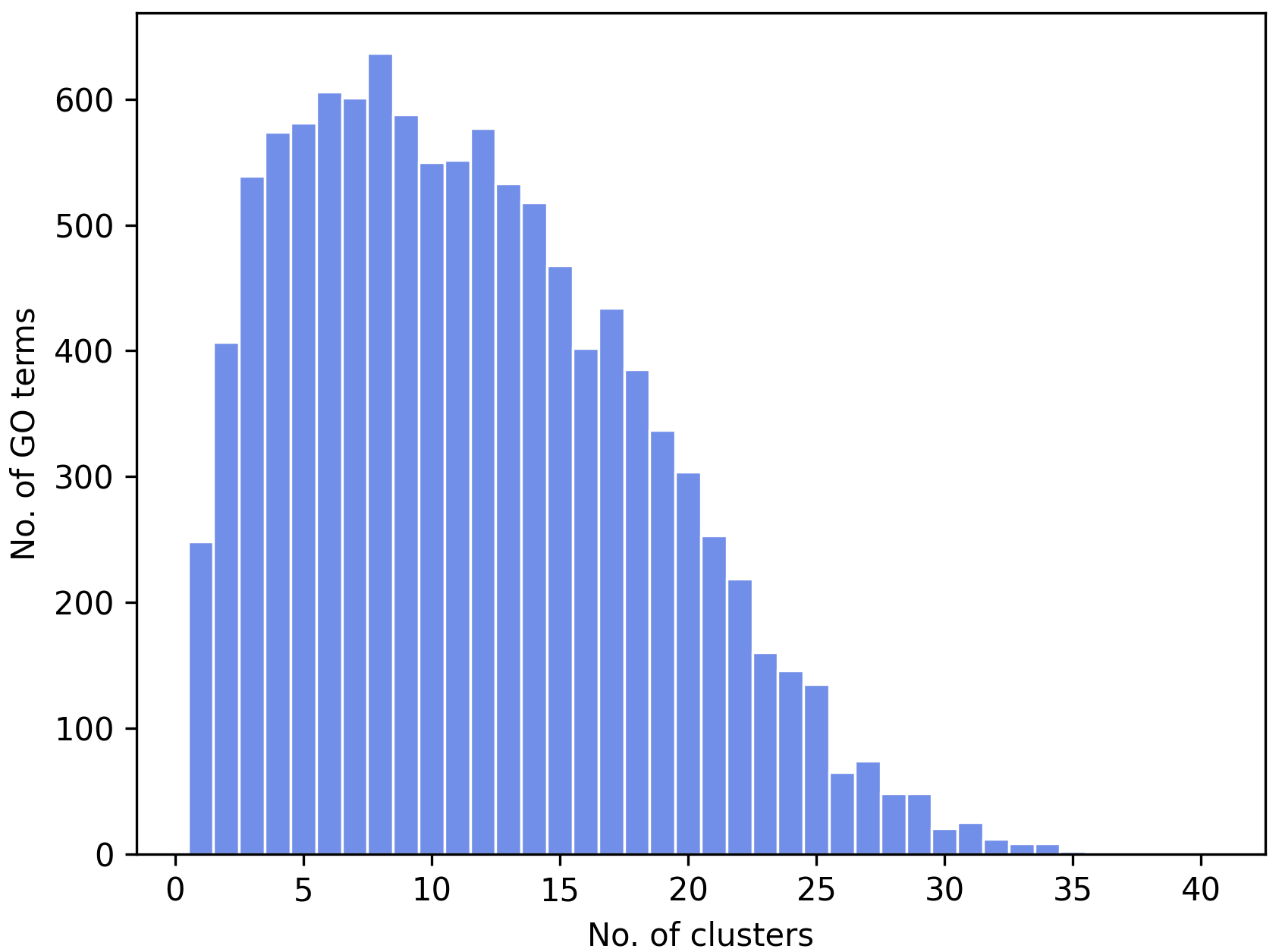


**Supp. Fig. 5: Histogram showing the number of GO terms (y-axis) that are enriched in a given number of** $\boldsymbol{G}_{\boldsymbol{2}}$ **clusters.** An enrichment analysis is done between every GO term and every $G_{2}$ cluster. We then compute how many GO terms (y-axis) are found to be enriched in different No. of clusters of SNPs (x-axis).


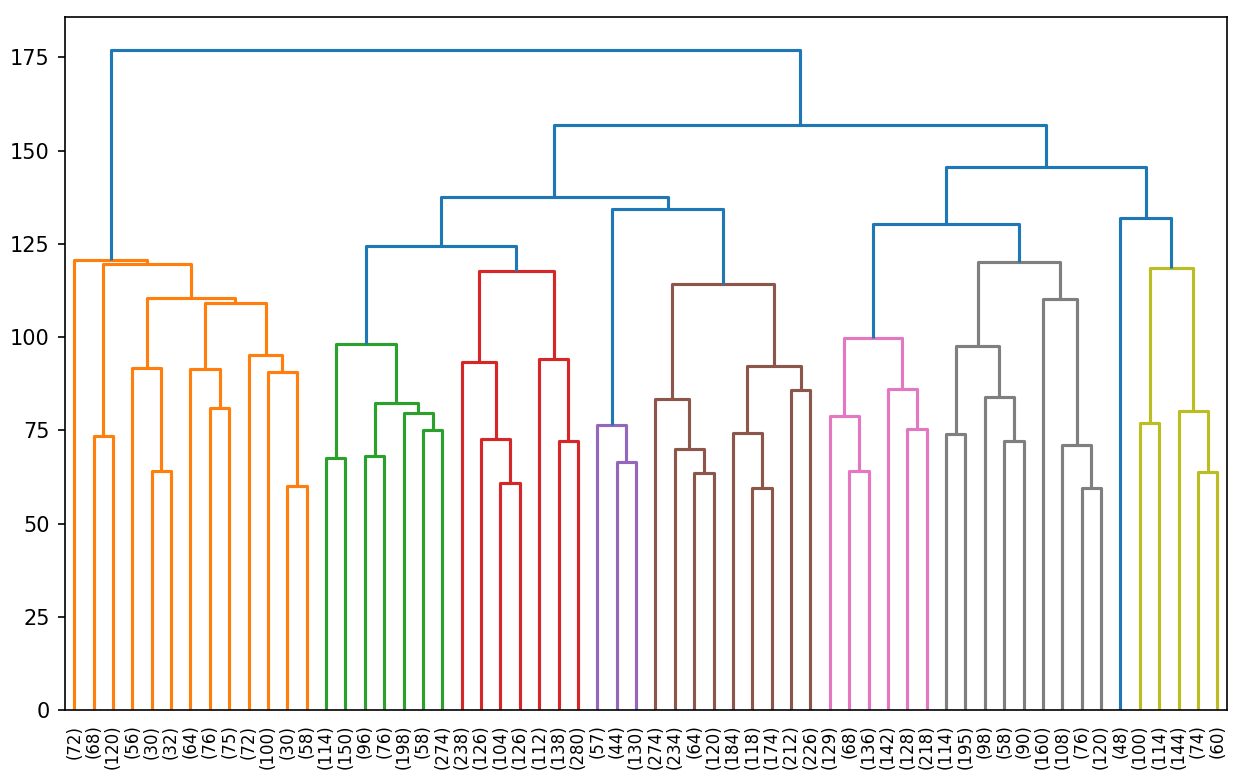


**Supp. Fig. 6: The dendrogram of the hierarchical clustering based on** $\boldsymbol{G}_{\boldsymbol{3}}$**.** Only the last 60 clusters formed in Ward’s linkage are shown and the size of every cluster is indicated in parentheses.


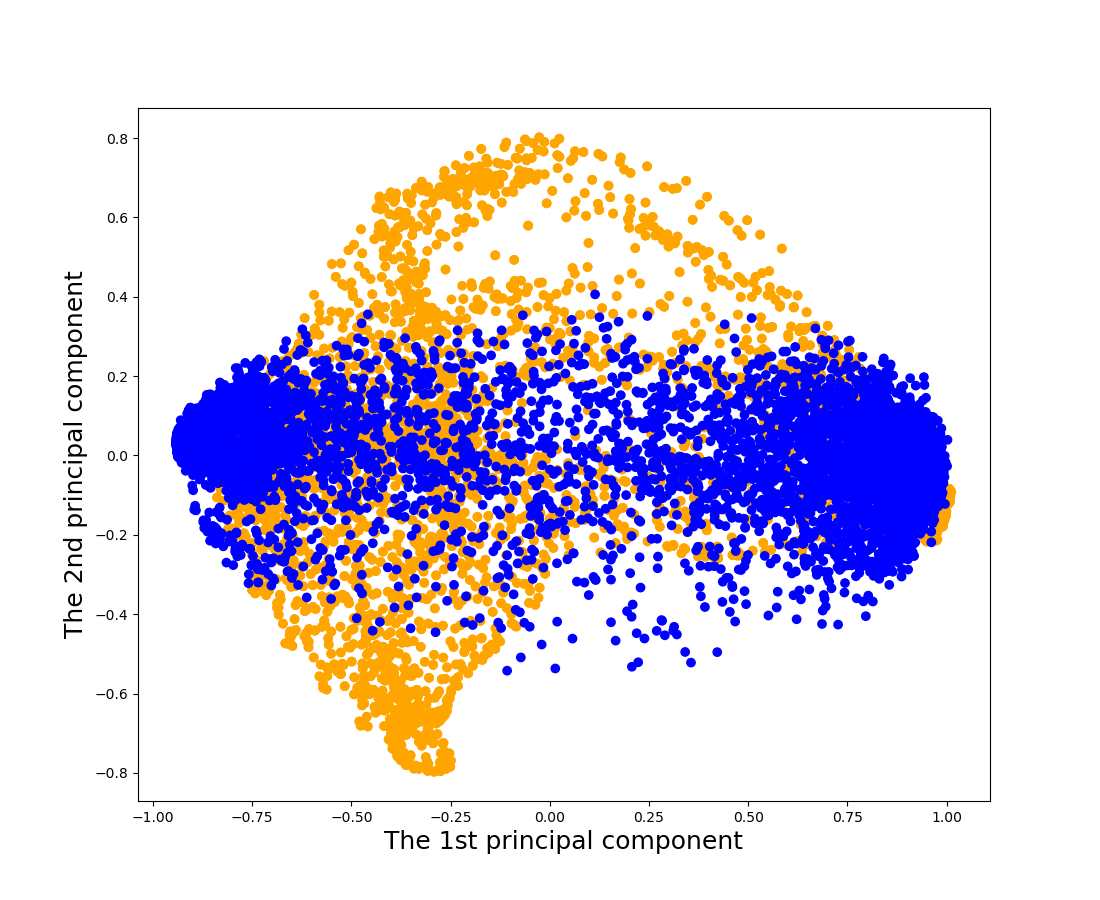


**Supp. Fig. 7: The scatter plot of SNPs (in blue) and facial landmarks (in orange) with the first two principal components.** The plot was created using principal component analysis (PCA) with cosine kernel on 7,160 randomly selected SNPs and all 7,160 facial landmarks in the space of $G_{1}$. The subsampling of SNPs is for balancing the two data types and the ease of visualization.


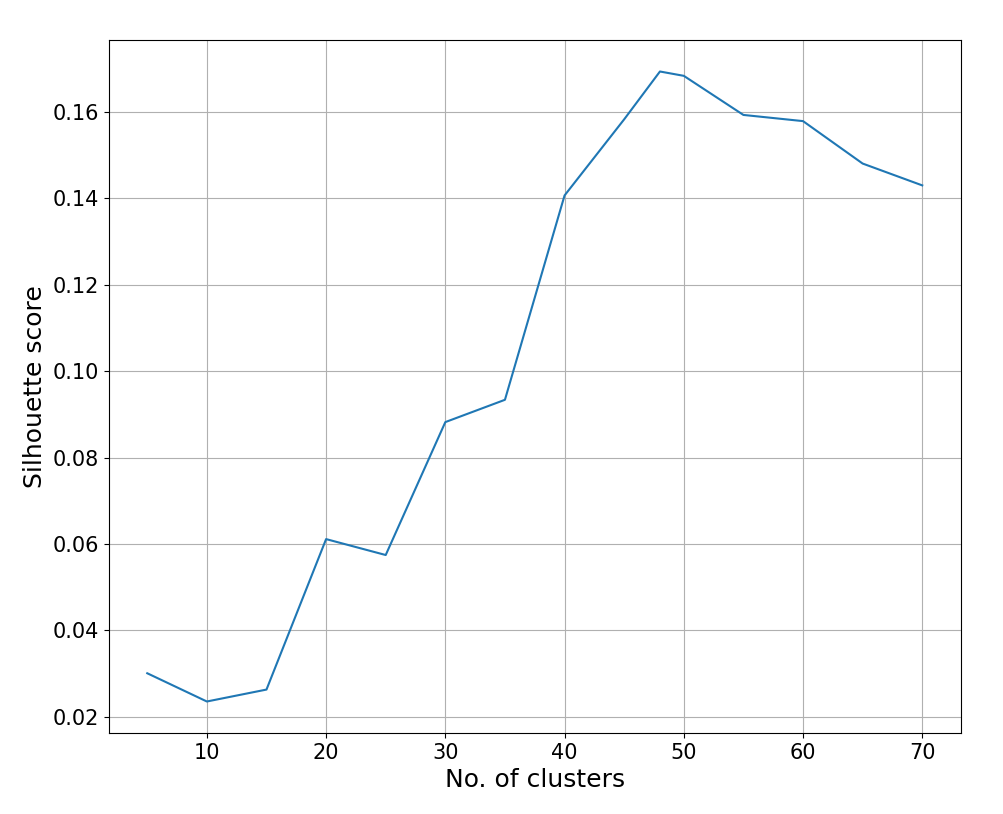


**Supp. Fig. 8: Silhouette score (y-axis) for different number of clusters (x-axis) based on** $\boldsymbol{k}$**-means clustering on** $\boldsymbol{G}_{\boldsymbol{1}}$**.**


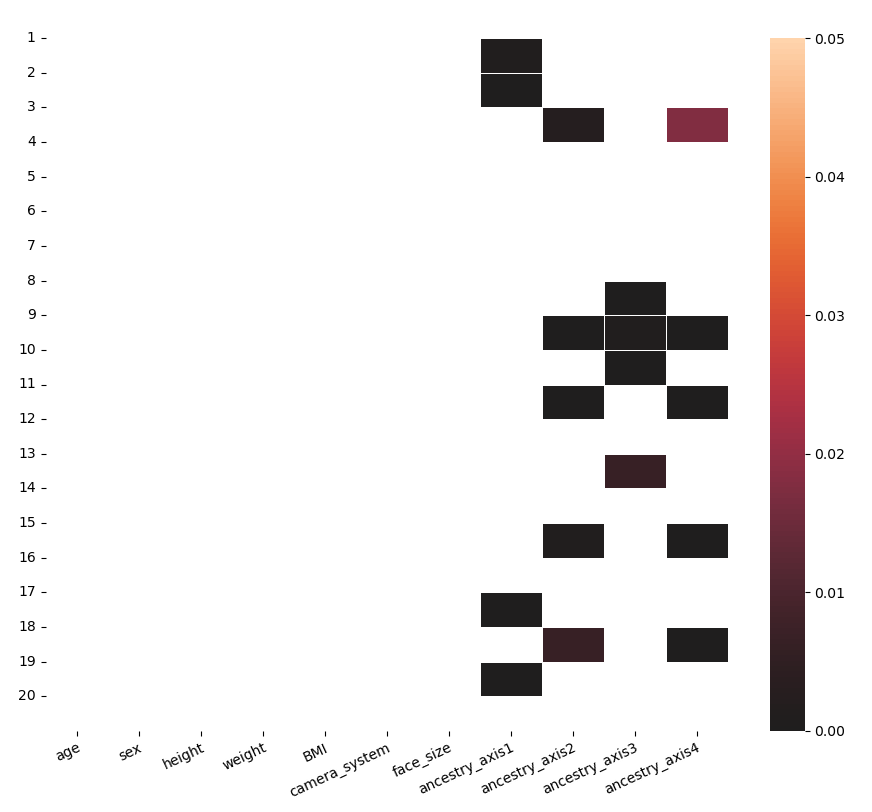


**Supp. Fig. 9: Heatmap of P-values for the F test between every unconfounded** $\boldsymbol{G}_{\boldsymbol{1}}$ **embedding vector and every confounder and ancestry axis.**


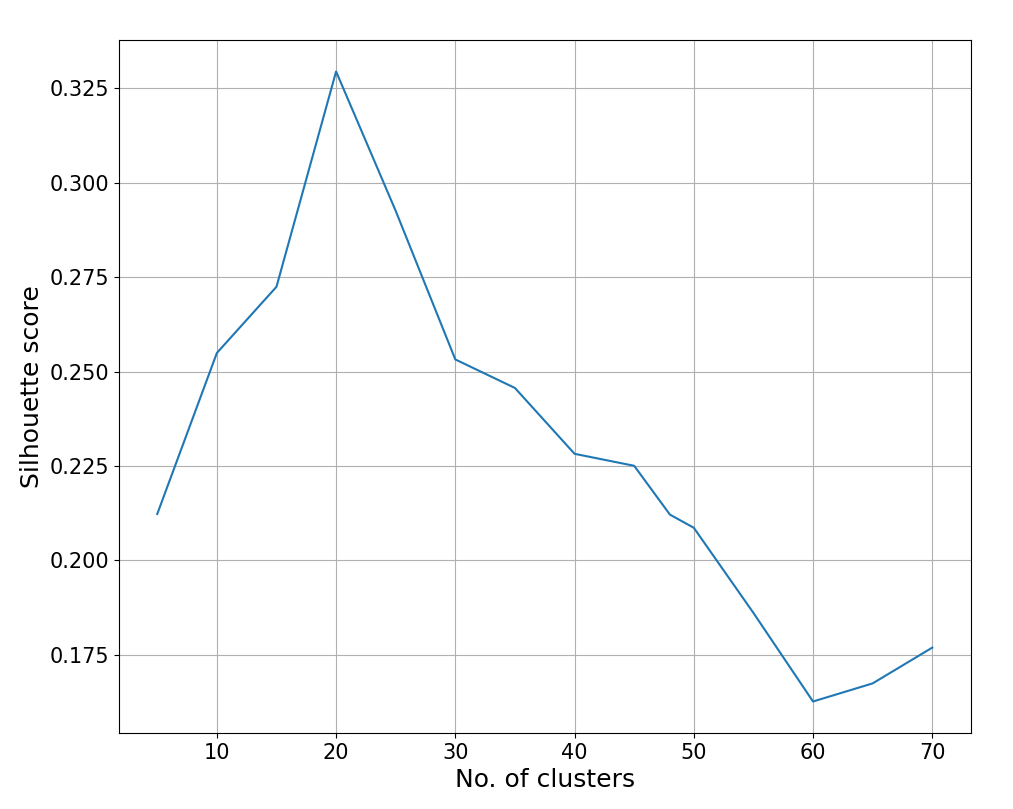


**Supp. Fig. 10: Silhouette score (y-axis) for different number of clusters (x-axis) based on** $\boldsymbol{k}$**-means clustering on the unconfounded** $\boldsymbol{G}_{\boldsymbol{1}}$**.** The unconfounded $G_{1}$ is composed of the 20 vectors of $G_{1}$ that are not significantly associated with any confounders.


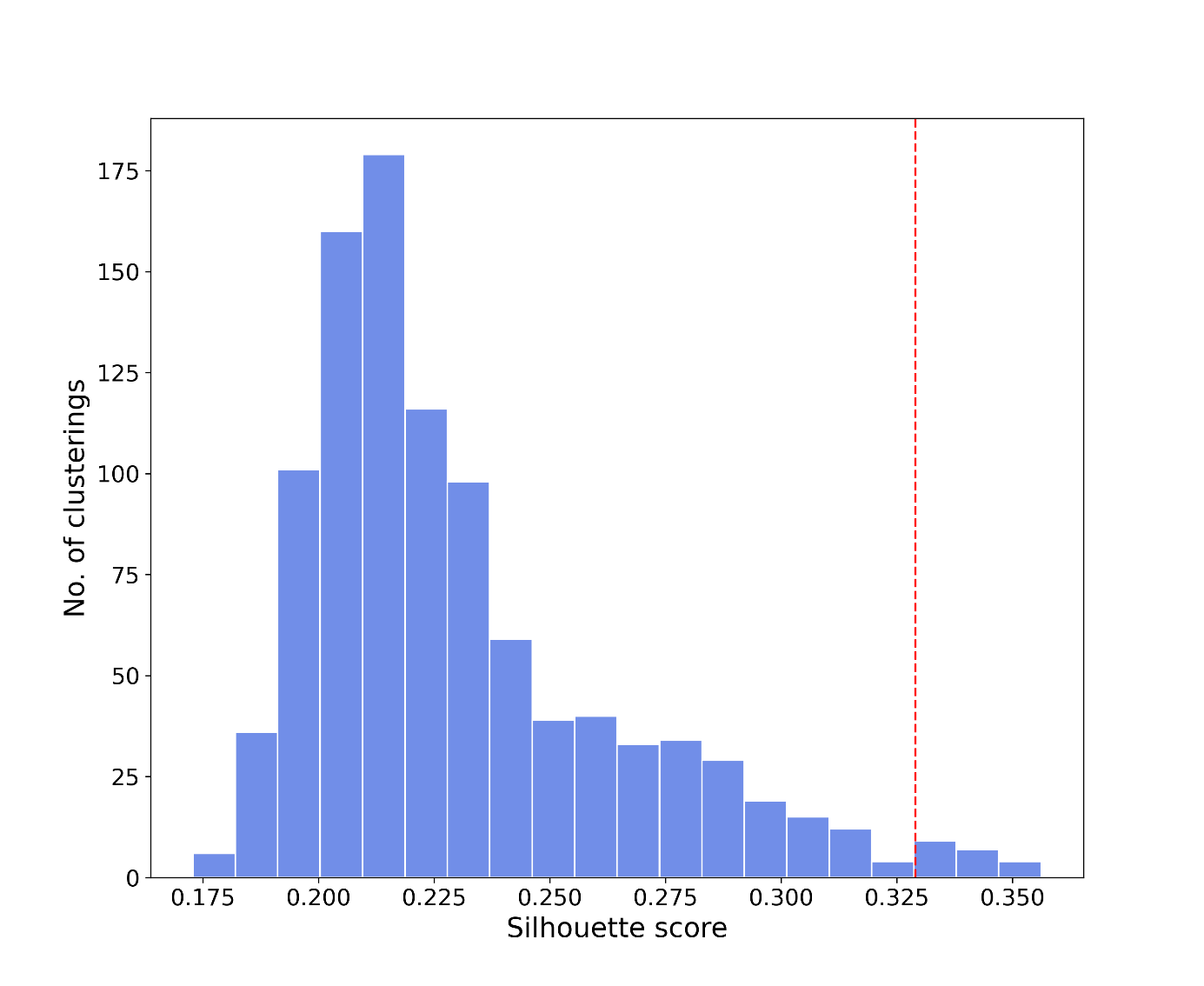


**Supp. Fig. 11: Histogram of Silhouette scores from 1000 repetitions of** $\boldsymbol{k}$**-means clustering based on 20 randomly sampled** $\boldsymbol{G}_{\boldsymbol{1}}$ **vectors.** The number of clusters for each repetition is the same (20). The red vertical line indicates the Silhouette score (0.329) of the clustering derived from 20 unconfounded $G_{1}$ vectors, also with 20 clusters.

| **Subgroup No.** | **Adj. P-value** | **Subgroup No.** | **Adj. P-value** |
| --- | --- | --- | --- |
| 1 | **4.73e-04** | 11 | **1.27e-03** |
| 2 | **1.70e-08** | 12 | 1.00 |
| 3 | **7.81e-06** | 13 | 0.130 |
| 4 | **3.75e-03** | 14 | 1.00 |
| 5 | **2.60e-03** | 15 | 0.200 |
| 6 | **3.90e-02** | 16 | 5.83e-02 |
| 7 | **7.99e-08** | 17 | **4.77e-02** |
| 8 | 1.00 | 18 | 9.83e-02 |
| 9 | 1.00 | 19 | 0.638 |
| 10 | **3.75e-03** | 20 | **6.94e-07** |

**Supp. Table 1: Adjusted log-likelihood ratio (LLR) P-values from logistic regression models for each population subgroup.** For each subgroup, a binary variable (whether an individual belongs to this subgroup or not) was created as the dependent variable against four ancestry axes (plus intercept), modelled by logistic regression with the ‘lbfgs’ solver. All LLR P-values have been corrected for multiple testing via the Benjamini-Hochberg (BH) procedure. Adjusted P-values lower than 0.05 (threshold for significance) are in bold.
